# Zebrafish larvae as a model for studying the impact of oral bacterial vesicles on tumor cell growth and metastasis

**DOI:** 10.1007/s13577-024-01114-6

**Published:** 2024-08-13

**Authors:** Marjut Metsäniitty, Saika Hasnat, Carina Öhman, Tuula Salo, Kari K. Eklund, Jan Oscarsson, Abdelhakim Salem

**Affiliations:** 1 Department of Oral and Maxillofacial Diseases, Clinicum, University of Helsinki, 00014 Helsinki, Finland; 2 Oral Microbiology, Department of Odontology, Umeå University, 90187 Umeå, Sweden; 3 Department of Rheumatology, University of Helsinki and Helsinki University Hospital, 00014 Helsinki, Finland; 4 Translational Immunology Research Program (TRIMM), Research Program Unit (RPU), University of Helsinki, 00014 Helsinki, Finland

**Keywords:** Cancer cell line, Extracellular vesicles, Oral bacteria, Zebrafish larvae, Oral cancer

## Abstract

Oral bacteria naturally secrete extracellular vesicles (EVs), which have attracted attention for their promising biomedical applications including cancer therapeutics. However, our understanding of EV impact on tumor progression is hampered by limited in vivo models. In this study, we propose a facile in vivo platform for assessing the effect of EVs isolated from different bacterial strains on oral cancer growth and dissemination using the larval zebrafish model. EVs were isolated from: wild-type *Aggregatibacter actinomycetemcomitans* and its mutant strains lacking the cytolethal distending toxin (CDT) or lipopolysaccharide (LPS) O-antigen; and wild-type *Porphyromonas gingivalis*. Cancer cells pretreated with EVs were xenotransplanted into zebrafish larvae, wherein tumor growth and metastasis were screened. We further assessed the preferential sites for the metastatic foci development. Interestingly, EVs from the CDT-lacking *A. actinomycetemcomitans* resulted in an increased tumor growth, whereas EVs lacking the lipopolysaccharide O-antigen reduced the metastasis rate. *P. gingivalis*-derived EVs showed no significant effects. Cancer cells pretreated with EVs from the mutant *A. actinomycetemcomitans* strains tended to metastasize less often to the head and tail compared to the controls. In sum, the proposed approach provided cost- and labor-effective yet efficient model for studying bacterial EVs in oral carcinogenesis, which can be easily extended for other cancer types. Furthermore, our results support the notion that these nanosized particles may represent promising targets in cancer therapeutics.

## Introduction

Oral squamous cell carcinoma (OSCC) is among the most common malignancies worldwide, accounting for more than 90% of oral cavity cancers [1, 2]. The majority of OSCC cases are diagnosed at locoregionally advanced stages, leading to high morbidity and mortality rates. Hence, the 5-year survival rate of these patients has remained stagnant at approximately 50% over the past decades [3, 4]. The main risk factors for OSCC are smoking, alcohol abuse and the consumption of tobacco products [5]. In addition, recent evidence suggests that oral microbiota may play a role in oral carcinogenesis [6]. Oral dysbiosis, an imbalance of oral bacteria, can promote various chronic inflammatory diseases including periodontitis, which has been linked to OSCC [7, 8]. On the contrary, some bacteria showed anti-tumorigenic effects and were associated with favorable prognostic outcomes [6]. Furthermore, oral microbiota was shown to differ between OSCC patients with and without lymph node metastasis [9]. Therefore, bacterial species and their role in cancer can vary across different individuals [10].

Oral bacteria actively secrete extracellular vesicles (EVs), which are important immunomodulators carrying multiple virulence factors [11]. Importantly, these nanosized particles have attracted attention for their biomedical applications such as vaccination and cancer therapy [12, 13]. However, despite the recent increasing interest in bacterial EVs, their role in cancer remains elusive with limited studies [14, 15]. EVs from the Gram-negative *Aggregatibacter actinomycetemcomitans* carry a variety of cargo, including the cytolethal distending toxin (CDT)—a genotoxin with DNase activity that has been implicated in head and neck cancers [16–19]. In addition, the immunomodulator lipopolysaccharide (LPS) is a major constituent of *A. actinomycetemcomitans* EVs and it has been suggested as a target in cancer therapy [20–22]. Importantly, loss of the LPS O-antigen significantly altered the pathogenic and immunostimulatory features of *A. actinomycetemcomitans* [23, 24]. Another Gram-negative anaerobic bacterium, *Porphyromonas gingivalis,* is one of the most studied periodontopathogens in OSCC, revealing mostly pro-tumorigenic effects [25]. Recently, we showed that EVs isolated from *A. actinomycetemcomitans* and *P. gingivalis* differentially influenced the behavior of OSCC cells in vitro [26]. Furthermore, *P. gingivalis*-derived EVs promoted OSCC cell migration and invasion in vitro [27]. However, research exploring the role of bacterial EVs in cancer is still limited, to our knowledge, with only three studies in OSCC to date [26–28].

In vivo studies exploring the bacterial role in cancer are currently conducted using patient-derived murine xenografts [28–32]. However, the utility of these models is dampened by cost, time, and labor challenges, thus hindering advancement in this new field. During recent years, zebrafish larvae have emerged as a favorite organism for wide-ranging studies of cancer [33–36]. Herein, we aimed to assess the utility of zebrafish larvae as a facile and rapid in vivo model for studying the influence of EVs from different *A. actinomycetemcomitans* strains and wild-type *P. gingivalis* on OSCC growth and metastasis.

## Materials and methods

### Bacterial strains and growth conditions

Four different strains of *A. actinomycetemcomitans* were used in this study (Table 1)*. A. actinomycetemcomitans* D7SS is a serotype a, naturally genetic competent, smooth-colony derivative of wild-type strain D7S, which is isolated from a patient with aggressive periodontitis [37]. D7SS *cdtABC* is a mutant derivative of D7SS created via a knockout method [38]. Hereafter, they are referred to as D7SS-WT, and D7SS-*cdt*, respectively. *A. actinomycetemcomitans* strains SA3138 [39] and SA3139 [39, 40] were recovered from a patient with periodontitis, and the latter strain naturally lacks LPS O-antigen [40]. Hereafter these are referred to as SA3138-WT, and SA3139-LPS-O, respectively. In addition, *P. gingivalis* ATCC 33277 (American Type Culture Collection) was used [41] (Table 1). Briefly, *A. actinomycetemcomitans* strains were cultured on blood agar plates (5% defibrinated horse blood, 5 mg hemin/l, 10 mg Vitamin K/l, Columbia agar base; Oxoid Ltd., Basingstoke, Hampshire, UK), in air supplemented with 5% CO_2_, at 37 °C. The D7SS strains were cultivated for 4 days and SA3138 and SA3139 for 5 days. *P. gingivalis* was cultured in an anaerobic environment (10% H_2_, 5% CO_2_, 85% N_2_) at 37 °C first on blood agar plates for 3 days and then for additional 48 h in liquid broth fastidious anaerobe agar (FAA; Neogen®, Heywood, UK). Bacterial procedures were conducted according to the guidelines of the local ethics committee at the Medical Faculty of Umeå University.

**Table 1:** Characteristics of bacterial extracellular vesicles

Bacterium	Strain	Source	EV protein concentration (mg/ml)*
*Aggregatibacter actinomycetemcomitans*	D7SS wild-type	Patient with periodontitis	1.987
*Aggregatibacter actinomycetemcomitans*	D7SS *cdtABC* mutant	Patient with periodontitis	1.258
*Aggregatibacter actinomycetemcomitans*	SA3138 wild-type	Patient with periodontitis	7.813
*Aggregatibacter actinomycetemcomitans*	SA3139 naturally lacking LPS O-antigen	Patient with periodontitis	8.732
*Porphyromonas gingivalis*	ATCC 33277	ATCC (Gingival sulcus)	2.132

*cdtABC* cytolethal distending toxin subunit A, B and C gene, *LPS* lipopolysaccharide, *ATCC American Type Culture Collection*

^*^Protein concentration of the vesicle samples was measured with NanoDrop 100 spectrophotometer (Thermo Fisher Scientific)

### EV isolation and analyses

The EV isolation was conducted by ultracentrifugation as recently reported [24, 42]. In brief, bacterial cells were harvested from agar plates and suspended in phosphate-buffered saline (PBS) or liquid broth. The optical density (OD) values of the 25 ml suspensions at 600 nm were: 0.76 (D7SS-WT), 0.56 (D7SS-*cdt*), 1.12 (SA3138-WT), 1.38 (SA3139-LPS-O) and 1.00 (*P. gingivalis)*. The number of agar plates used for harvesting the bacterial cells was 5 (D7SS-*cdt)*, 10 (D7SS-WT, SA3138-WT and SA3139-LPS-O). The suspensions were centrifuged at 12.096×*g* for 30 min at 4 °C in a JA-25.50 rotor (Beckman Instruments Inc.). Supernatants were filtered through syringe filters (0.45 and 0.2 µm, Filtropur, Sarstedt) and centrifuged at 85.000×*g* for 2 h at 4 °C in a 70 Ti rotor (Beckman Instruments Inc.). Then pellets were washed with PBS twice (85.000×*g* for 2 h at 4 °C in a Sw60 Ti rotor (Beckman Instruments Inc.) and suspended in PBS. Absence of contamination was tested by cultivating small EV sample aliquots on blood agar plates in air supplemented with 5% CO_2_ at 37 °C for 3 days. EV protein concentration was determined by NanoDrop 100 spectrophotometer (Thermo Fisher Scientific) and further analyzed by nanoparticle tracking analysis software Zetaview (Particle Metrix, Germany). A protein gel electrophoresis was conducted with Pierce™ Silver Stain Kit (Thermo Fisher Scientific) according to the manufacturer’s instructions to visualize EV proteins. We used the Criterion™ TGX™ Precast Gels and Precision Plus Protein™ Standard All Blue (Bio-Rad). Images were taken with ChemiDoc™ MP imaging system.

### Cancer cell lines and growth conditions

To investigate tumor cell metastasis in vivo, we used the highly metastatic OSCC cell line HSC-3 (JCRB Cell Bank, Japan). Cancer cells were cultured in 1:1 DMEM/F-12 medium which was supplemented with 10% heat-inactivated fetal bovine serum (Gibco), penicillin–streptomycin (Gibco), 250 ng/mL amphotericin B (Sigma-Aldrich, St. Louis, MO, USA), 50 µg/mL ascorbic acid (AppliChem, Chicago, IL, USA), and 0.4 µg/mL hydrocortisone (Sigma-Aldrich, St. Louis, MO, USA). Cell maintenance and incubations were done at 37 °C, 5% CO_2_ concentration and 95% relative humidity unless otherwise indicated. HSC-3 cell line was authenticated by Technology Centre, Institute for Molecular Medicine Finland FIMM, University of Helsinki.

### Zebrafish larvae xenograft

The effect of bacterial EVs on OSCC tumor area and metastasis in vivo was investigated using zebrafish larvae [36]. HSC-3 cells (4 × 10^6^) were challenged with EVs (10 µg/ml) for 12 h. The control cells were cultured in the same DMEM medium but without EVs. The selected EV concentration was based on recent studies [27, 28, 43, 44]. The next day, cells were dyed with CellTrace™ Far Red Cell Proliferation Kit (Thermo Fisher Scientific, Cat. No. C34564) prior to implantation into the zebrafish larvae via microinjection. All zebrafish larvae wild-type (AB strain) were used at two-day post-fertilization (dpf). Fish were dechorionated and anesthetized with 0.04% Tricaine before microinjection to the perivitelline space, mimicking a subcutaneous injection in mouse model, with a 4 nl suspension of HSC-3 cells (1500 cells/4 nl/larva). Fish microinjection and experiments were conducted at the Zebrafish Unit (University of Helsinki) and approved by the ethical permission from the regional state administrative agency (ESAVI/13139/04.10.05/2017). After microinjection, the larvae were transferred to a 24-well plate containing 1000 µl fresh embryonic medium (Merck) and stored at 34 °C. Each group included 19–25 larvae, divided into wells with a maximum of five fish per well. After 72 h, the zebrafish larvae were fixed with 4% paraformaldehyde overnight. The next day, they were washed twice with 1% PBS and mounted on slides with SlowFade™ Gold Antifade Mountant (Thermo Fisher Scientific, S36837).

### Imaging and image analysis

The mounted zebrafish larvae were imaged using a Leica Thunder Imager 3D Cell Culture microscope with Plan Fluotar 10×/0.32NA objective at the Biomedicum Imaging Unit, University of Helsinki, Finland. The tumor area was measured using Fiji ImageJ software (Wayne Rasband, National Institute of Health, Bethesda, MD, USA).

### Statistical analyses

Statistical analyses were performed with GraphPad Prism Software version 9.4.1 (San Diego, California, USA). The Grubbs` test was used to identify and remove the outlier values, which were considered significant when *p* < 0.01. The analysis included groups of 18–25 fish per condition which were pooled together. As the variation in tumor area between experiments was significant, the two-way ANOVA with Dunnett`s multiple comparison test was used to determine statistical significance. Differences in metastasis between each condition and control were calculated with Fisher`s exact test with Bonferroni correction. Statistical significance was set to *p* < 0.05, * indicates *p*-values < 0.05. Data are represented as mean ± standard deviation (SD) or as quartiles with range from minimum to maximum with median and mean. The experiments were repeated three times independently.

## Results

### Zebrafish larvae survival

To our knowledge, this is the first study to evaluate the effect of bacterial EVs on OSCC cells using zebrafish larvae model. A total of 389 fish were included in the final analysis from the following testing groups: no-treatment control; D7SS-WT; D7SS-*cdt*; SA3138-WT; SA3139-LPS-O and *P. gingivalis*. The survival rate of the zebrafish larvae following xenotransplantation was 98.73%, with only a few fish died (n = 5) before mounting.

### Tumor area

**Fig. 1:**
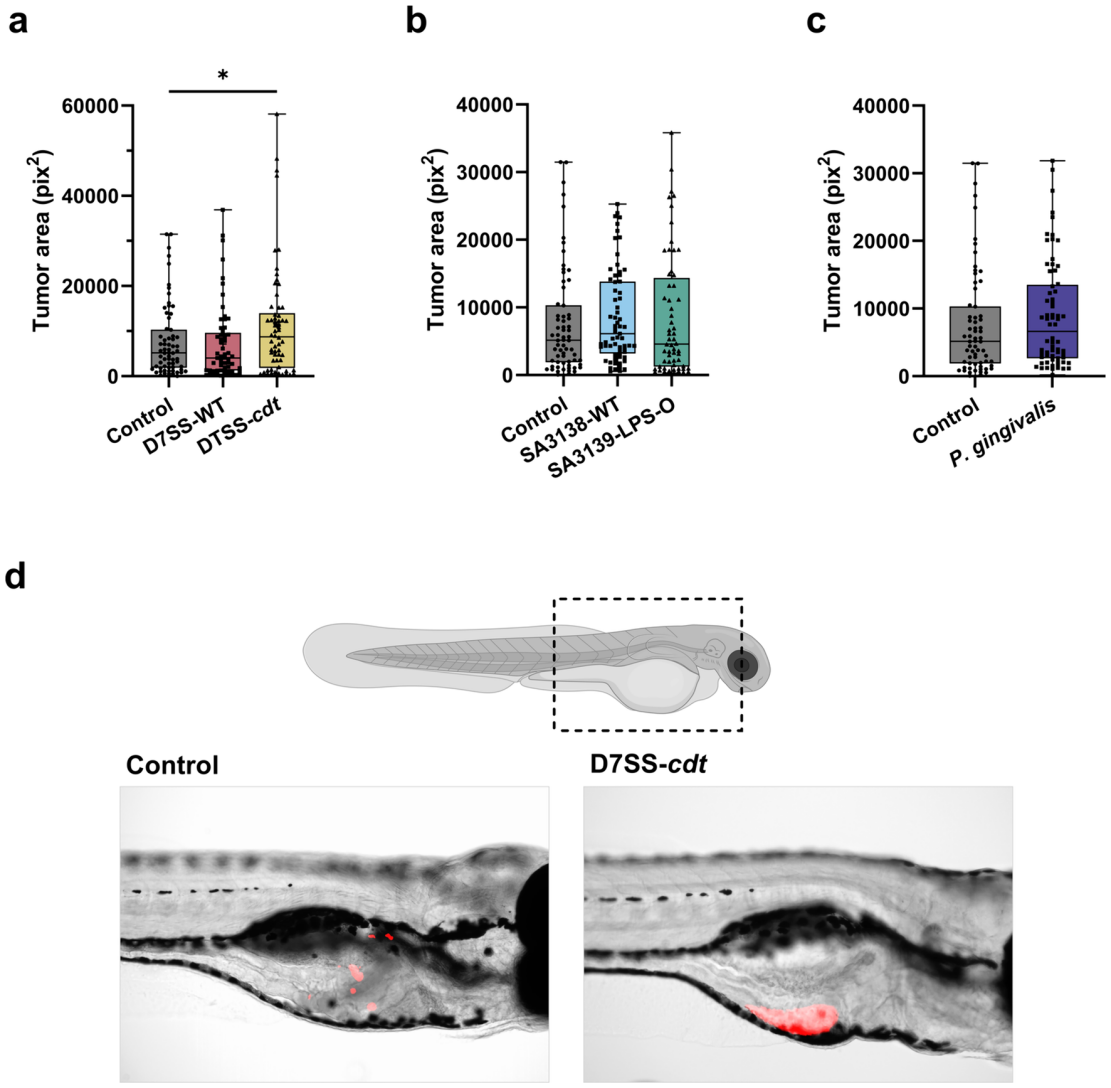
Tumor area of HSC-3 cells pretreated with bacterial extracellular vesicles (EVs; 10 µg/ml) from: *A. actinomycetemcomitans* D7SS-WT (wild-type), D7SS-*cdt* (lacking the cytolethal distending toxin, CDT), SA3138-WT (wild-type), SA3139-LPS-O (lacking the lipopolysaccharide (LPS) O-antigen), and *P. gingivalis* (wild-type)*.* **a** HSC-3 cells pretreated with D7SS-*cdt*-derived EVs formed larger tumors than control cells (*p* < 0.05). **b** No statistically significant changes were seen in the tumor area of cells pretreated with EVs from SA3138-WT, SA3139-LPS-O or **c** EVs from *P. gingivalis*. **d** Representative images of tumors formed by control cells and cells pretreated with D7SS-*cdt* EVs. Red areas represent tumor cells. *p < 0.05. Values are shown as minimum to maximum with all individual values. All experiments were repeated independently three times

Tumor area was calculated in pix^2^ using Fiji ImageJ. We compared the effect of EVs from CDT-expressing *A. actinomycetemcomitans* on tumor area in vivo. Interestingly, while there was no significant difference between D7SS-WT-derived EVs and control, EVs from D7SS-*cdt* strain increased the tumor area significantly (*p* < 0.05; Fig. 1a, d). The effect of LPS O-antigen in *A. actinomycetemcomitans* EVs on OSCC tumor size was also tested using the wild- type strain SA3138-WT and SA3139-LPS-O strain which is naturally lacking LPS O-antigen. Both strains showed variations in the tumor area compared to the control and hence no statistically significant differences were noted (Fig. 1b). To compare *A. actinomycetemcomitans* strains to another common periodontopathogen, we tested EVs from wild-type *P. gingivalis* which, however, did not show any effects on tumor area compared to control (Fig. 1c).

### Tumor cell metastasis

**Fig. 2:**
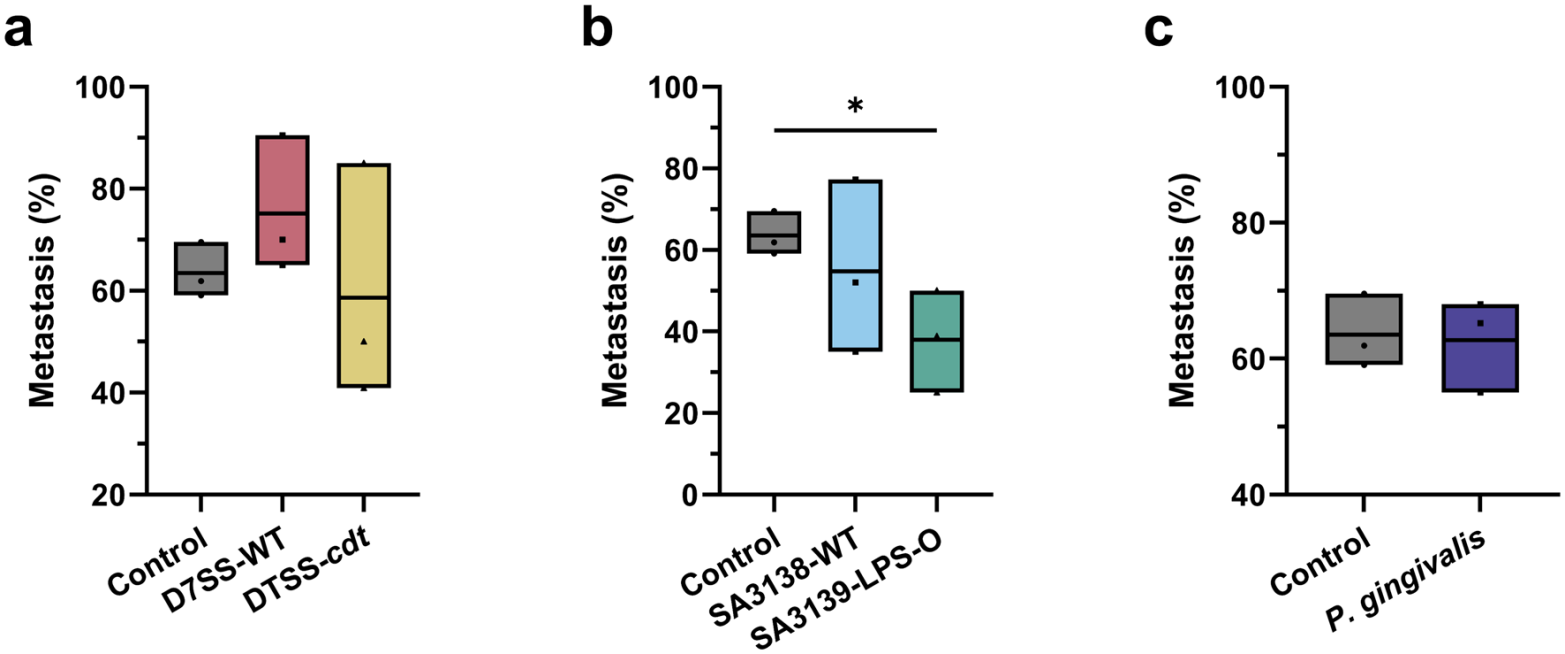
Metastasis of HSC-3 cells pretreated with bacterial extracellular vesicles (EVs; 10 µg/ml) from: *A. actinomycetemcomitans* D7SS-WT (wild-type), D7SS-*cdt* (lacking the cytolethal distending toxin, CDT), SA3138-WT (wild-type), SA3139-LPS-O (lacking the lipopolysaccharide (LPS) O-antigen), and *P. gingivalis* (wild-type)*.* **a** Metastasis rate was not significantly affected by pretreatment with EVs from D7SS-WT and D7SS-*cdt* strains. **b** HSC-3 cells pretreated with SA3139-LPS-O-derived EVs had significantly lower metastatic rate than control cells. **c** No statistically significant changes were seen in the metastasis rates of HSC-3 cells pretreated with *P. gingivalis* EVs compared to controls. * *p* < 0.05. Values are shown as mean values from each experiment and line at mean. All experiments were repeated independently three times

Next, we analyzed the tumor metastasis in zebrafish. Metastasis was analyzed by counting the proportion of fish with tumor cells metastasized outside the perivitelline area, i.e., head or tail, in each treatment group. A cut-off value of ≥1 cell outside the perivitelline area was considered as metastasis. Percentual averages ± SD from the three experiments were: control, 63.52 ± 5.42%; D7SS-WT, 75.16 ± 13.50%; D7SS-*cdt*, 58.64 ± 23.28; SA3138-WT, 54.76 ± 29.90%; SA3139-LPS-O, 34.63 ± 6.85%; and *P. gingivalis,* 62.74 ± 5.42%. The CDT expression in *A. actinomycetemcomitans* EVs did not influence tumor cell metastasis, and neither D7SS-WT nor D7SS-*cdt* were significantly different from the control (Fig. 2a). EVs from the wild-type *A. actinomycetemcomitans* strain SA3138-WT did not affect metastasis, but EVs from SA3139-LPS-O strain lacking LPS O-antigen significantly reduced metastasis compared to control (*P* < 0.05; Fig. 2b). We did not observe difference in metastasis between OSCC cells treated with *P. gingivalis*-derived EVs and control cells (Fig. 2c).

### Site of tumor dissemination

**Fig. 3:**
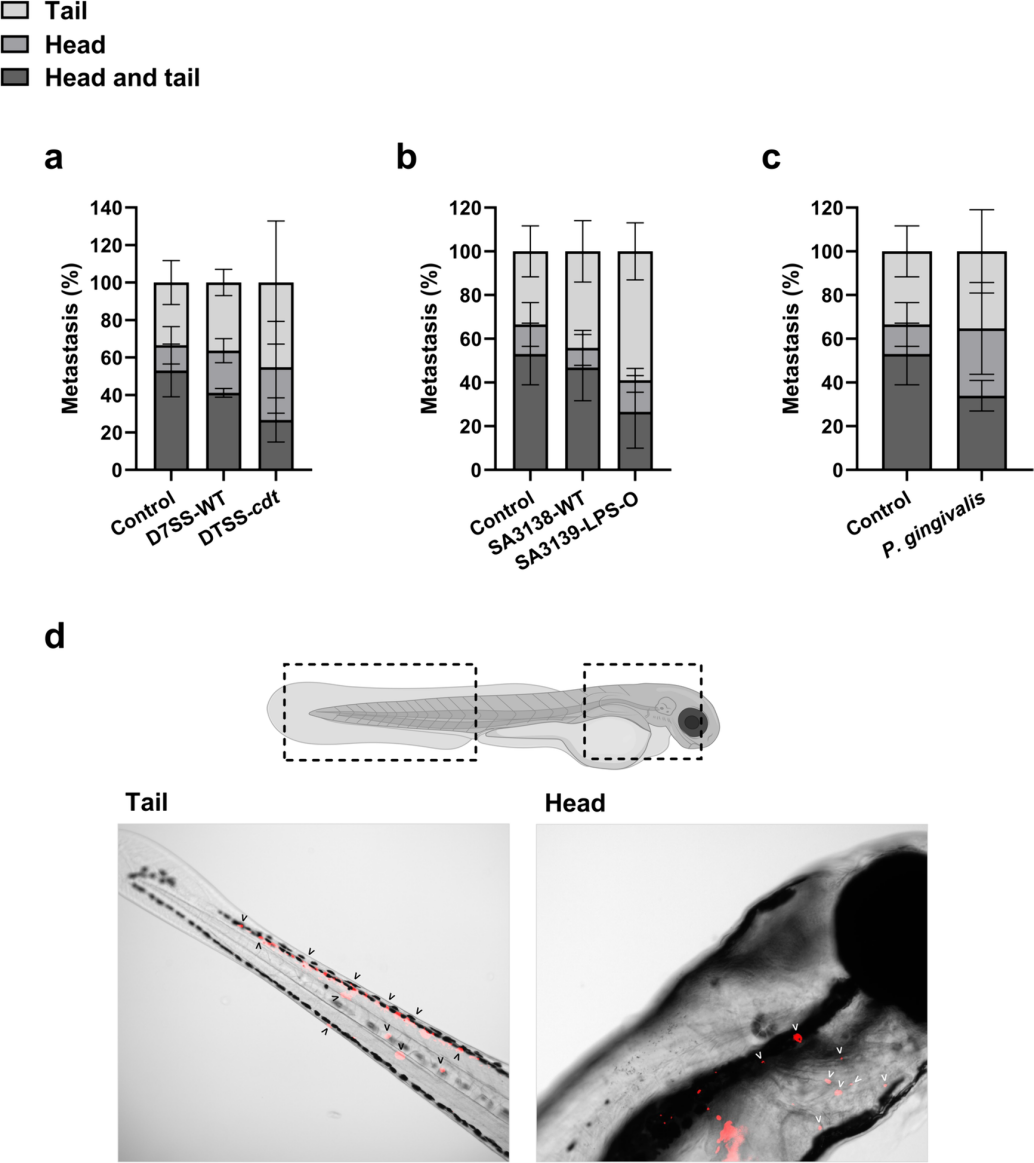
Preferential dissemination sites of the metastatic tumor cells in zebrafish larvae. HSC-3 cells were treated with bacterial extracellular vesicles (EVs; 10 µg/ml) from: *A. actinomycetemcomitans* D7SS-WT (wild-type), D7SS-*cdt* (lacking the cytolethal distending toxin, CDT), SA3138-WT (wild-type), SA3139-LPS-O (lacking the lipopolysaccharide (LPS) O-antigen), and *P. gingivalis* (wild-type)*.* A trend of lower metastasis rate to both head and tail was noted in cells pretreated with EVs from **a** *A. actinomycetemcomitans* D7SS-*cdt*, **b** *A. actinomycetemcomitans* SA3139-LPS-O and **c** *P. gingivalis*, although the differences were not statistically significant. **d** Demonstrative images of metastasis in head and tail of zebrafish larvae. Red areas represent tumor cells. Values are shown as mean ± SD. All experiments were repeated independently three times

In addition to analyzing metastasis, we further screened whether the metastatic foci were detected in the zebrafish head, tail, or in both head and tail. The differences compared to control were not statistically significant (Fig. 3a–c). Though, an interesting pattern was seen that, OSCC cells pretreated with EVs from the mutant *A. actinomycetemcomitans* strains D7SS-*cdt* and SA3139-LPS-O tended to metastasize less often to the head and tail compared to the control. The trend was consistent in all three experiments. Percentual averages ± SD from the three experiments showed that among all metastasis cases, control tumors metastasized more often to head and tail (53.04 ± 14.07%), clearly more than cells pretreated with D7SS-*cdt* EVs (26.62 ± 11.80%) and SA3139-LPS-O EVs (19.91 ± 23.24%) (Fig. 3a, b). Cells pretreated with *P. gingivalis* EVs metastasized a little more often to head and tail (33.93 ± 6.97%) but less than the controls, showing a consistent, though non-significant, trend in all three experiments (Fig. 3c).

## Discussion

The present study is the first to investigate the interactions between bacterial EVs and OSCC in vivo using zebrafish larvae. Interestingly, we reported that pretreatment with EVs from *A. actinomycetemcomitans* D7SS-*cdt* strain resulted in an increased tumor area, while those from the SA3139-LPS-O strain showed lower metastasis rates. No significant changes were observed in cells pre-challenged with *P. gingivalis* EVs.

Previously, several studies utilized zebrafish larvae in bacterial research [45–48]. For instance, zebrafish larvae were used to study the effect of *P. gingivalis* on vascular permeability and systemic dissemination [45–47]. However, to our knowledge, this model has not been employed for studying the influence of bacterial EVs on cancer cell growth and metastasis to date. Currently, the interactions between bacterial EVs and cancer are studied in vivo using patient-derived murine xenografts [28, 44, 49, 50]. In these studies, EVs were administered for immunization prior cancer cell implantation in a model of murine melanoma [50]; intravenously after implantation of murine mammary, adenocarcinoma and melanoma cells [44]; subcutaneously after implantation of murine lung carcinoma cells [49]; or intratumorally in OSCC tumors formed by human tongue cancer cells [28]. In zebrafish larvae, the studied compounds can be administered via immersion (i.e. from embryonic medium), microinjection or through pretreatment of cancer cells prior implantation [51]. We opt herein for the latter approach and pretreated the OSCC cells with the EVs, based on previous in vitro and in vivo studies [27, 28, 52]. Alternatively, in a previous study, *Escherichia coli* cells were added to the embryonic medium to study hepatic and breast cancer in zebrafish model [48]. Adding EVs to the embryonic medium could also be considered, however, they might not be easily immersed due to the hydrophobic nature of EVs [51].

*A. actinomycetemcomitans* is the only known oral bacterium that produces CDT [53], which is delivered into host cells via EVs [42]. CDT from *A. actinomycetemcomitans* showed antitumorigenic potential in leukemia, oral, prostate and lung cancers [16, 17, 19, 54–56] but CDT has also been suggested to promote carcinogenesis via DNA damage [18, 57]. We showed that HSC-3 cells pretreated with EVs from the CDT-lacking strain formed larger tumors in vivo. Interestingly, our recent in vitro findings revealed that HSC-3 cell proliferation was not affected by EVs from the D7SS-*cdt* strain; while only the wild strain EVs significantly reduced the proliferation of the metastatic tumor cells [26]. However, despite their larger sizes, they did not exhibit a higher metastasis rate. In this regard, OSCC tumor size does not always correlate with metastasis [58] but rather with the depth of invasion and tumor budding [58, 59].

The periodontopathogen *P. gingivalis* has been shown to mainly promote pro-tumorigenic effects in OSCC [25, 26, 30, 60]. Importantly, *P. gingivalis* promoted key features for metastasis including oral epithelial cell “stemness” and epithelial-mesenchymal transition (EMT) [61–64]. Furthermore, *P. gingivalis* and its EVs promoted OSCC cell migration and invasion [27, 65, 66]. In agreement, *P. gingivalis* promoted OSCC tumor growth [31, 32] and metastasis [29] in mice. Our findings did not reveal any significant effects of *P. gingivalis* EVs on tumor growth or metastasis. The reason is not clear; however, this encourages further studies using different EV doses and treatment durations.

Metastasis is the main cause of morbidity and cancer-related deaths in OSCC patients [67, 68]. Unlike its wild-type equivalent, EVs from the *A. actinomycetemcomitans* strain lacking O-antigen in LPS showed reduced dissemination of the highly metastatic HSC-3 cells. The *A. actinomycetemcomitans* O-antigen is an immunodominant, serotype-specific polysaccharide of LPS [69]. One of the key benefits of using zebrafish larvae in cancer research is their compatibility with xenograft transplantation. During the first 30 dpf, the fish larvae have only innate immune cells and lack an adaptive immune response, thus eliminating concerns of immune rejection in larval xenografts [70, 71]. In this context, considering the immunodeficient nature of this model, it is improbable that the findings are due to LPS-mediated immune modulation as commonly seen in other cancer studies [72]. Of note, only LPS from periodontopathogens, but not LPS from commensal bacteria, influenced oral cancer cells directly in vitro [32]. Thus, it is logical to assume that the structural variation of EVs, mediated by different LPS structure, could affect cell metastasis. LPS consists of hydrophobic lipid A, hydrophilic core polysaccharide and hydrophilic O-antigen [73]. Lack of O-antigen reduces the hydrophilicity of EVs and possibly affects EV properties in vivo.

In conclusion, we explored the feasibility of zebrafish larvae as a simple yet efficient model for studying bacterial EVs and OSCC in vivo. Our findings revealed both pro- and anti-tumorigenic effects of EVs from *A. actinomycetemcomitans* strains, depending in part on their expression of CDT and LPS O-antigen. Given the promising utility of bacterial EVs as potential therapeutic targets in cancer, we encourage further research on these nanosized molecules using the zebrafish larvae, which overcome many limitations associated with the traditional murine models.

## Data Availability

Data is provided within the manuscript and also available from the lead authors upon a reasonable request.
